# Normal, abnormal, and inconclusive: has the ultrasound pattern of healthy cervical lymph nodes been defined?

**DOI:** 10.1590/0100-3984.2016.49.5e3

**Published:** 2016

**Authors:** Ana Célia Baptista Koifman

**Affiliations:** 1 Adjunct Professor of Radiology at the Universidade Federal do Estado do Rio de Janeiro (Unirio) and at the Universidade do Estado do Rio de Janeiro (UERJ), Rio de Janeiro, RJ, Brazil. E-mail: anaceliak@gmail.com.

Normality can be conceptualized as the rule, as the commonplace, or even as what is not unusual or different, that does not stand out. Therefore, it can be understood as a comparative parameter. Over the course of human history, criteria have been developed to facilitate our understanding of the world. Human judgments regarding the external reality are curious. Detectives deceive themselves, at times, by believing the words of chronic liars and of murderers. The binomials technique/experience and rationality/intuition reduce the chance of error in the perception of the facts; so is it also in medicine. The number of successes in judgments grows in partnership with knowledge and technology, and we, radiologists, are the human way for the translation of images into diagnoses. The standards of normality can vary between people, within the same person (i.e., among different regions of the body), and among imaging methods. Recognizing what is normal avoids unnecessary costs, delays in diagnosis and treatment, and families' anxiety.

The assessment of head and neck injuries by imaging methods has been the focus of a number of recent studies in the radiology literature of Brazil^(1,6)^. The article authored by Ogassavara et al.^(7)^, published in the previous issue of **Radiologia Brasileira**, brought to light the normal morphological aspects of superficial lymph nodes of the neck in adult patients, on gray-scale ultrasound, which take on greater importance due to the rarity and brevity of descriptions in the literature. The lymph node is an encapsulated unit, composed of lymphoid lobules, surrounded by lymph-filled sinuses, which displays inherent variation. The number of lobules varies according to the size and, within the same lymph node, lobules show different levels of immune activity and do not always present an uniform aspect^(8)^. Each lymph node lobule has three parts: the cortex (or superficial cortex), the paracortex (or deep cortex), and medulla. Ultrasound enables to identify a normal central hilum, which is hyperechoic due to sound reflection interfaces between blood vessels and fat, clearly differentiated from the cortex and paracortex, which are hypoechoic. In addition, several other parameters, including cortical thickness, morphology (concentric or eccentric), size, and shape (sphericity index), can be evaluated by this widely available, low-cost, portable method that uses no ionizing radiation^(8,9)^. Ogassavara et al.^(7)^ showed that there is considerable variation in lymph node size between normal patients and among cervical regions within the same patient. Although ultrasound analysis encompasses various characteristics, size is considered important in the morphological evaluation and might represent the starting point for the investigation in the majority of cases.

Eyes and hands are our basic guides during the application of this method, whose image should always be given weight and judged in conjunction with clinical data, together with the results of any previous examinations (at baseline or before), as well as data obtained during follow-up. The closer we get to diagnostic "perfection", the more wisdom and experience is required in order to read the results of an examination correctly. Further studies, evaluating a greater number of variables and including other age groups, such as children and the elderly, would be most welcome. After all, normal is our reference for the absence of disease. "Diagnosing" normal is good for everyone's health!
